# The Knowledge‐Attitude Paradox in Analgesic Self‐Medication Among University Students in Ecuador: A Cross‐Sectional Study and Predictive Modeling Analysis

**DOI:** 10.1002/puh2.70169

**Published:** 2025-11-20

**Authors:** Sonia Argote, Doménica Bucheli, Ashel Páez, Camila Rovayo, Daniel Freire, Pablo Pila, José Daniel Sánchez

**Affiliations:** ^1^ Facultad de Ciencias de la Salud Universidad Tecnológica Indoamérica Quito Ecuador

**Keywords:** analgesics, attitudes, behavioral intervention, Ecuador, knowledge, predictive modeling, public health, self‐medication, university students

## Abstract

**Objective:**

To evaluate the prevalence and determinants of analgesic self‐medication among university students in Ecuador, with a focus on the interplay between pharmacological knowledge and personal attitudes.

**Methods:**

A cross‐sectional analytical study was conducted with 422 students at Universidad Tecnologica Indoamerica (April–August 2025), selected via stratified random sampling. A validated, structured questionnaire assessed knowledge, attitudes, and practices. Multivariable logistic regression and random forest algorithms were employed to identify key predictors and their relative importance.

**Results:**

The prevalence of analgesic self‐medication was exceptionally high at 87.5%. Random forest analysis identified attitude as the primary predictor of this behavior (relative importance = 0.252), followed by academic year and pharmacological knowledge. A significant “knowledge–attitude paradox” was observed, wherein health sciences students, despite possessing superior pharmacological knowledge, exhibited the highest rates of self‐medication. The predictive model demonstrated excellent discrimination (AUC = 0.81).

**Conclusion:**

The high prevalence of self‐medication, a finding consistent with emerging international data, necessitates a fundamental paradigm shift in public health interventions. Educational strategies must evolve beyond simple information dissemination to incorporate behavioral science principles aimed at modifying attitudes and risk perceptions, particularly during the formative early years of university education.

## 1 Introduction

Self‐medication, defined as the use of therapeutic agents to treat self‐diagnosed symptoms or conditions without professional medical supervision, represents a multifaceted global public health phenomenon with profound implications for patient safety, antimicrobial resistance, and healthcare resource allocation [1]. The World Health Organization (WHO) acknowledges self‐medication as a component of responsible self‐care when practiced appropriately but emphasizes the critical need for adequate regulation, education, and monitoring to minimize potential adverse outcomes. The practice is driven by numerous factors, including difficulties in accessing healthcare, poor drug regulatory enforcement, and the widespread availability of over‐the‐counter (OTC) medications [2].

### 1.1 Global Prevalence and Patterns

University students constitute a particularly vulnerable demographic for problematic self‐medication practices, with reported prevalence rates exhibiting substantial global variation. Rates range from 38.5% in some Ethiopian cohorts to alarmingly high levels in other regions [3]. This study's finding of an 87.5% prevalence in Ecuador is remarkably congruent with recent European data, where a study among Croatian dental students reported a prevalence of 91.7% [4]. This parallel between a Latin American and a European context suggests that the drivers of this behavior may transcend specific healthcare systems and cultural settings. Indeed, the literature documents a consistently high prevalence among health sciences students globally, with rates of 92.4% in Iraq, 95.0.

### 1.2 The Knowledge–Attitude–Practice (KAP) Framework and Its Paradox

The KAP theoretical framework has been extensively employed to understand health behaviors, postulating that knowledge influences attitudes, which subsequently determine practices. However, emerging evidence suggests this linear relationship is often oversimplified, particularly in educated populations. A critical “knowledge–attitude paradox” has been observed, where individuals with extensive medical knowledge, such as health science students, do not necessarily demonstrate safer self‐medication behaviors [5, 6]. Recent evidence from Simunovic et al. provides compelling quantitative support for this paradox, finding that as dental students progressed academically, both their pharmacological knowledge and their frequency of self‐medication increased concurrently [4]. This suggests that the core issue is not a failure of knowledge to influence practice directly, but rather a failure of knowledge to appropriately shape the underlying attitudes and risk perceptions that are the true drivers of behavior.

### 1.3 Latin American Context and Research Gap

Despite the substantial body of international literature, comprehensive data from Latin America, particularly Ecuador, remain markedly limited. Existing regional studies have begun to address this gap, but a more comprehensive analysis incorporating advanced predictive modeling is essential for dissecting the specific determinants influencing self‐medication practices within the unique Ecuadorian healthcare and cultural context [7].

### 1.4 Study Objectives

This investigation aims to address the identified research gap through four primary objectives: (1) to determine the prevalence and patterns of analgesic self‐medication among university students at Universidad Tecnologica Indoamerica; (2) to identify and quantify key predictive factors using advanced statistical modeling approaches; (3) to develop a robust predictive model to assess the relative importance of these determinants; and (4) to provide evidence‐based recommendations for targeted public health interventions. Understanding these factors is crucial for developing effective, culturally appropriate interventions to promote responsible self‐medication and improve student health outcomes.

## 2 Materials and Methods

### 2.1 Study Design and Setting

A cross‐sectional analytical study was conducted between April and August 2025 among undergraduate students at Universidad Tecnologica Indoamerica, a private comprehensive university in Quito, Ecuador. The institution enrolls approximately 3200 students across diverse faculties, including health sciences, engineering, law and political science, and social sciences.

### 2.2 Sample Size Calculation and Sampling Methodology

The sample size was calculated using the formula for cross‐sectional studies in finite populations:

$$NZ^{2}p\left(1\;-\;p\right)$$

(1) $$n=d^{2}\left(N\;-\;1\right)+Z^{2}p\left(1\;-\;p\right)$$

where *N* = 3200 (total population), *Z* = 1.96 (95% confidence level), *p* = 0.70 (expected prevalence based on pilot data), and *d* = 0.05 (margin of error). The calculated minimum sample size was 384. To account for potential nonresponse, the sample was increased by 10% to 422 students. A stratified random sampling methodology was employed, with stratification by faculty and academic year, to ensure proportional representation.

### 2.3 Inclusion and Exclusion Criteria

**Inclusion criteria**: Currently enrolled undergraduate students, aged 18 years or older, who provided written informed consent.

**Exclusion criteria**: Students in distance learning programs, those with questionnaires having >20% missing data, and participants who withdrew consent.

### 2.4 Data Collection Instrument

A structured, self‐administered questionnaire was developed on the basis of previously validated instruments and adapted for the local context [8, 9]. The instrument underwent a multiphase validation process, including content validation by a panel of five experts, pilot testing with 30 students, and reliability assessment, which yielded strong internal consistency (Cronbach's *α* = 0.78 for knowledge, 0.82 for attitudes, and 0.75 for practices scales). The final questionnaire comprised four sections: (1) sociodemographic characteristics (8 items); (2) knowledge assessment (15 items, score range 0–20); (3) attitudes evaluation using a 5‐point Likert scale (10 items, score range 0–25); and (4) practices assessment (8 items, score range 0–15).

### 2.5 Data Collection Procedure

Four trained research assistants collected data during regular class hours, following a standardized protocol that emphasized voluntary participation and ensured confidentiality. Written informed consent was obtained from all participants.

### 2.6 Operational Definitions

**Self‐medication**: use of any analgesic medication without a healthcare provider's prescription within the 6 months preceding the survey.

**Knowledge score categories**: poor (0–10 points), fair (11–15 points), and good (16–20 points).

**Attitude score categories**: negative (0–10 points), neutral (11–15 points), and positive (16–25 points).

### 2.7 Statistical Analysis

Analyses were performed using SPSS version 28.0 and R version 4.3.0. The analysis included descriptive statistics, bivariate tests (*χ*2, *t*‐tests, Mann–Whitney *U*), and Pearson's correlation. A multivariable logistic regression model was constructed using backward elimination to identify independent predictors. A random forest algorithm (1000 trees) was implemented for feature importance analysis, using the mean decrease in Gini impurity to rank predictors [10]. Model performance was evaluated using the area under the receiver operating characteristic curve (AUC), the Hosmer–Lemeshow test, and 10‐fold cross‐validation. A *p* value <0.05 was considered statistically significant. The study adheres to the STROBE guidelines.

## 3 Results

### 3.1 Participant Characteristics and Response Rate

A total of 422 students completed the questionnaire, achieving a 95.3% response rate (422/443). The mean age of participants was 21.4 ± 2.8 years, and 58.3% (*n* = 246) were female. Students were proportionally distributed across academic years and faculties (Table 1).

**TABLE 1:** Sociodemographic characteristics and self‐medication patterns (*n* = 422).

Characteristic	*n* (%)	95% CI demographics
Age (years), mean ± SD	21.4 ± 2.8	21.1–21.7
Female gender	246 (58.3)	53.4–63.1
**Academic characteristics**		
First year	99 (23.5)	19.5–27.8
Second year	110 (26.1)	21.9–30.6
Third year	107 (25.4)	21.2–29.9
Fourth year+	106 (25.0)	20.8–29.5
Health sciences faculty	138 (32.7)	28.2–37.5
Engineering faculty	120 (28.4)	24.2–33.0
Law and political sciences faculty	102 (24.2)	20.1–28.6
Social sciences faculty	62 (14.7)	11.4–18.5
**Self‐medication patterns**		
Overall prevalence	369 (87.5)	84.1–90.3
Frequent users^a^	148 (35.0)	30.5–39.8
Paracetamol use	285 (67.5)	62.8–72.0
Ibuprofen use	110 (26.1)	21.9–30.6
Internet as information source	128 (30.3)	25.9–35.0

^a^ Defined as reporting self‐medication as “frequent” or “very frequent.”

### 3.2 Prevalence and Patterns of Analgesic Self‐Medication

The overall prevalence of analgesic self‐medication was 87.5% (95% CI: 84.1%–90.3%; *n* = 369). The most frequently used analgesics were paracetamol (77.2%) and ibuprofen (29.8%). The primary indications were headache (82.4%), menstrual pain (43.6%), and muscle pain (38.2%). Information sources were varied, including internet resources (30.2%), pharmacists (25.1%), and family/friends (20.3%).

### 3.3 KAP Assessment

The mean knowledge score was 12.6 ± 4.2 (out of 20), with health sciences students scoring significantly higher than their peers from other faculties (14.8 ± 3.5 vs. 11.2 ± 4.1, *p*<0.001). Table 2 details this disparity across knowledge domains. The mean attitude score was 17.3 ± 5.1 (out of 25), indicating generally favorable attitudes toward self‐medication, with no significant difference between faculties (*p* = 0.186).

**TABLE 2:** Knowledge components by faculty (mean scores ± SD).

Knowledge domain	Health sci.	Engineering	Law and Political Science	Social sci.
Analgesic types	4.1 ± 1.2	3.6 ± 1.1	3.4 ± 1.3	3.3 ± 1.2
Side effects	3.8 ± 1.2	2.6 ± 1.1	2.4 ± 1.0	2.3 ± 1.1
Maximum dosage	4.2 ± 1.1	2.5 ± 1.3	2.3 ± 1.2	2.1 ± 1.1
Contraindications	3.9 ± 1.0	2.4 ± 1.1	2.1 ± 1.0	2.0 ± 1.0
Drug interactions	3.8 ± 1.1	2.1 ± 1.0	1.9 ± 0.9	1.8 ± 0.9
**Total score**	**14.8** ± **3.5**	**11.2** ± **4.1**	**10.1** ± **3.8**	**9.5** ± **3.6**

*Note:* All comparisons between health sciences and other faculties: *p*<l0.001.

### 3.4 Correlation Analysis

A strong significant positive correlation was found between attitudes and practices (*r* = 0.524, *p*<0.001), indicating that more favorable attitudes were associated with more frequent self‐medication. A moderate correlation existed between knowledge and attitudes (*r* = 0.314, *p*<0.001), but the correlation between knowledge and practices was weak and not statistically significant (*r* = 0.118, *p* = 0.183).

### 3.5 Predictive Modeling and Feature Importance

**FIGURE 1:**
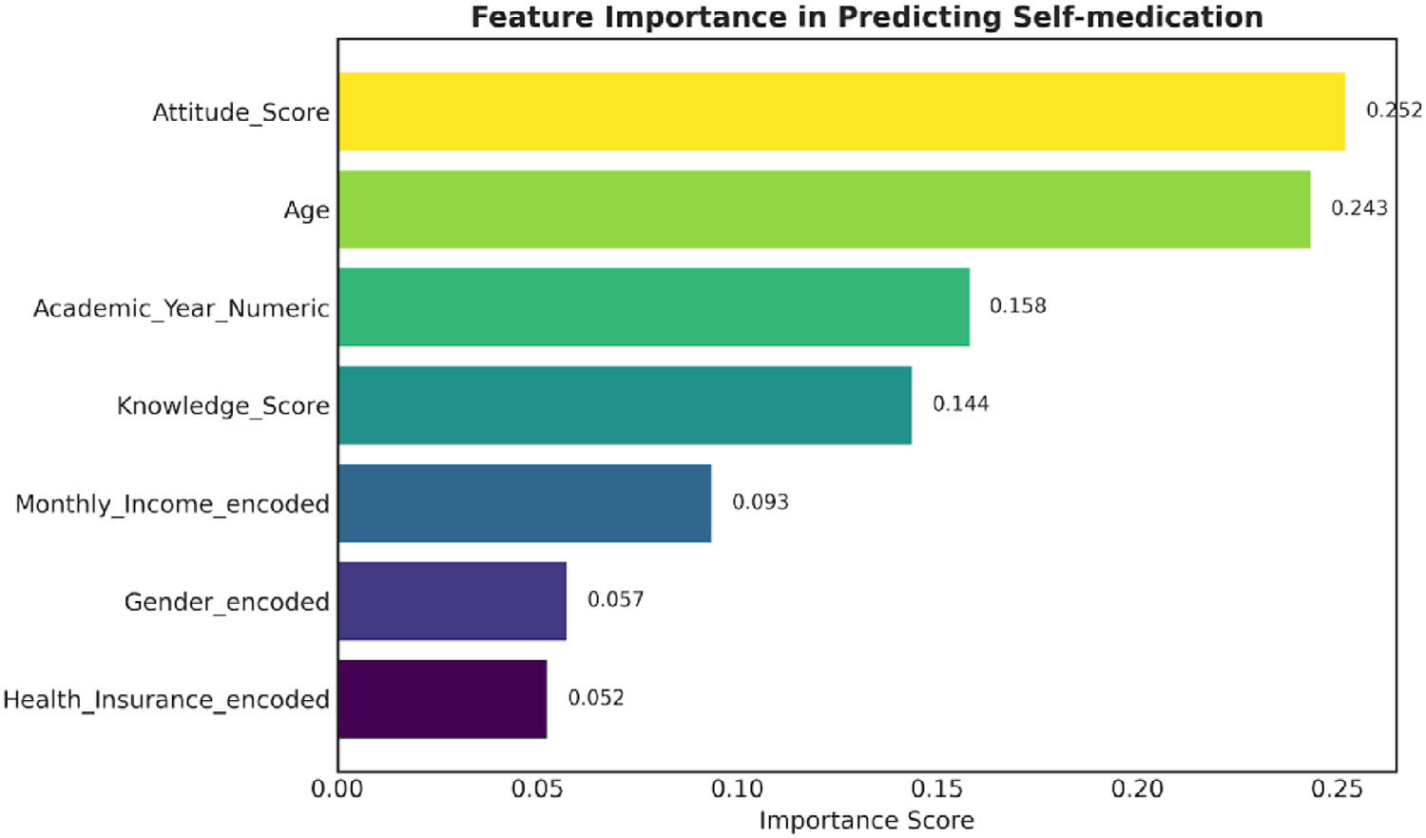
Feature importance in predicting analgesic self‐medication. Random forest analysis identified attitudes as the most important predictor (relative importance = 0.252), followed by academic year (0.243). Pharmacological knowledge ranked third (0.158), demonstrating lower predictive power than attitudinal and temporal factors.

The multivariable logistic regression model confirmed that attitudes and academic year were significant predictors of self‐medication (Table 3). The random forest analysis provided a clearer hierarchy of predictor importance, ranking attitudes as the most influential factor (relative importance = 0.252), followed by academic year (0.243) and pharmacological knowledge (0.158), as shown in Figure 1.

**TABLE 3:** Multivariable logistic regression analysis of self‐medication predictors.

Variable	OR	95% CI	*p* value
Attitudes (per point increase)	2.34	1.87–2.93	<<0.001
*Academic year (ref.: 1st year)*			
2nd year	1.52	0.88–2.62	0.134
3rd year	2.12	1.20–3.74	0.010
4th+ year	1.97	1.10–3.55	0.023
Knowledge (per point increase)	1.22	0.98–1.52	0.073
*Faculty (ref.: social sciences)*			
Health sciences	1.18	0.92–1.51	0.185
Engineering	1.15	0.89–1.49	0.279
Law and political sciences	1.10	0.84–1.44	0.508

*Note:* Model Nagelkerke *R* ^2^ = 0.298.

Abbreviations: CI, confidence interval; OR, odds ratio.

### 3.6 Model Performance and Validation

The predictive model demonstrated excellent performance, with an AUC of 0.81 (95% CI: 0.76–0.86), indicating strong discriminatory power. The model was well‐calibrated (Hosmer–Lemeshow test, *p* = 0.439) and showed good internal validity (10‐fold cross‐validation AUC = 0.79 ± 0.04).

### 3.7 The Knowledge–Attitude Paradox

**FIGURE 2:**
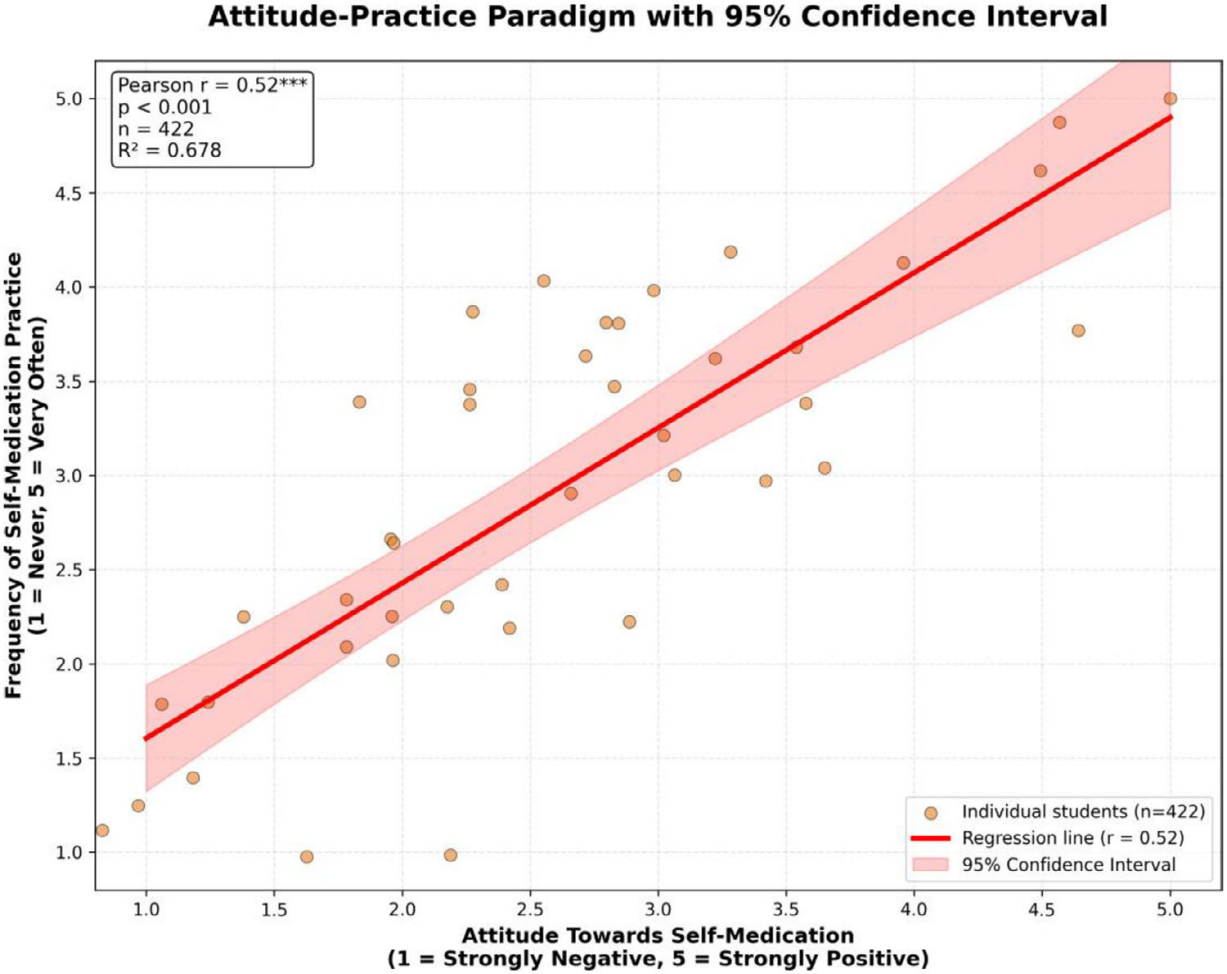
Attitude–practice paradigm in analgesic self‐medication. The scatterplot reveals a strong positive linear relationship (Pearson *r* = 0.524, *p*<l0.001) between attitudes toward self‐medication and the frequency of self‐medication practices. This demonstrates that attitudes are a powerful predictor of behavior.

A striking finding was the disconnect between knowledge and practice. Despite having significantly superior pharmacological knowledge (Table 2), health sciences students reported the highest prevalence of self‐medication (89.7%) compared to engineering (85.3%), aw and political sciences (83.9%), and social sciences (80.0%) students (*p* = 0.023). This paradox is further supported by the strong attitude–practice correlation (*r* = 0.524) versus the non‐significant knowledge–practice correlation (*r* = 0.118) (Figure 2).

## 4 Discussion

### 4.1 Principal Findings in a Global Context

This study reveals an exceptionally high prevalence of analgesic self‐medication (87.5%) among Ecuadorian university students, a rate that is not only higher than many regional estimates but also ranks among the highest documented globally. It approaches the upper limits seen in high‐prevalence regions like Nepal (81.9%) and Brazil (98.8%) and is remarkably congruent with the 91.7% prevalence recently reported among dental students in Croatia [4, 11]. This positions the issue as a significant public health concern in Ecuador. The predominance of paracetamol (77.2%) in the Ecuadorian cohort and ibuprofen (70%) in the Croatian cohort aligns with international patterns, reflecting their widespread availability and perceived safety, while also suggesting potential influences of local drug marketing or cultural preferences [4].

### 4.2 The Primacy of Attitudes: A Challenge to Conventional Health Education

The most significant finding is the identification of attitudes as the primary determinant of self‐medication behavior, demonstrating substantially greater predictive power than pharmacological knowledge. This result fundamentally challenges the conventional, knowledge‐centric approach to health education. It aligns strongly with established behavioral science frameworks, such as the Theory of Planned Behavior and the Health Belief Model, which posit that attitudes, perceived norms, and beliefs about susceptibility and benefits are more potent drivers of action than factual knowledge alone. The data suggest that students’ perceptions of self‐medication as a convenient, effective, and low‐risk solution for minor ailments override their technical understanding of pharmacology. This indicates a necessary shift in intervention strategy, from a paternalistic model focused on information transfer to a collaborative one aimed at understanding and reshaping the underlying belief systems that govern behavior [12].

### 4.3 Deconstructing the Knowledge–Attitude Paradox: International Validation and a Systemic Educational Failure

The “knowledge–attitude paradox” observed in this study—where health sciences students, despite superior knowledge, exhibit the highest rates of self‐medication—is a deeply concerning phenomenon that resonates with findings from international studies. This is not a true paradox but rather a predictable outcome of an educational model that prioritizes technical competence over the metacognitive skills required for safe self‐care. Recent research from Croatia provides powerful, quantitative, external validation for this concept. Simunovic and colleagues found a statistically significant positive correlation between a higher year of study and both superior knowledge (*r* = 0.2115) and more frequent self‐medication (*r* = 0.2102) [4]. This finding provides a direct, quantitative parallel to this study's observation that health sciences students—the most knowledgeable cohort—reported the highest prevalence of self‐medication (89.7%). This phenomenon is further triangulated by a study in Bahrain, which found that senior medical students possessed “better knowledge” yet “practiced self‐medication more often” than their junior counterparts [13]. The convergence of this evidence from Ecuador, Croatia, and Bahrain transforms a single‐study observation into a documented, international phenomenon.

Several mechanisms likely contribute to this disconnect. First, enhanced knowledge can foster an overconfidence bias, creating an inflated sense of competence in self‐diagnosis and treatment that leads to a downplaying of risks [14]. Students may self‐assess their knowledge as sufficient to bypass professional consultation, a cognitive bias that specialized education fails to mitigate. Second, the clinical training environment may inadvertently normalize the frequent use of medication for minor ailments, shaping attitudes through a process of professional socialization where self‐medication is modeled as an efficient coping strategy. Finally, there may be a misaligned risk perception, where students rationalize risky behaviors by separating their personal actions from their professional knowledge. They are taught how to treat patients but are not adequately trained to apply the same principles of caution to themselves. The acquisition of knowledge is not a neutral event; it appears to actively recalibrate the student's risk‐assessment framework, often in a direction that favors convenience over caution. This highlights a critical gap in health education: curricula excel at imparting pharmacological facts but often fail to integrate behavioral science principles that would equip future health professionals with the self‐awareness and risk‐assessment skills needed to be responsible stewards of their own health.

### 4.4 The University Trajectory as a Critical Window for Intervention

The finding that self‐medication prevalence increases with academic year is consistent with international research and points to a pattern of behavioral entrenchment over time [4, 13]. This temporal trend identifies the early university years as a critical window for intervention before these behaviors become habitual. The transition to university life is a formative period for establishing health habits and is often accompanied by escalating psychosocial stressors like academic pressure and financial concerns, which can drive maladaptive coping behaviors like self‐medication. Interventions timed to coincide with this period, particularly in the first and second years, are likely to have the greatest impact.

### 4.5 Strengths and Limitations

This study's strengths include its robust sample size, high response rate, use of a validated instrument, and sophisticated statistical methodology incorporating both regression and machine learning to elucidate predictor importance. However, limitations must be acknowledged. The cross‐sectional design precludes causal inference. Self‐reported data are subject to recall and social desirability biases. Although the single‐institution setting may limit generalizability, the striking congruence of the key findings with recent international studies, such as the one from Croatia, enhances the external validity and potential applicability of the conclusions [4]. Future research should employ longitudinal designs to track the evolution of KAP and cognitive biases over time, conduct multi‐center studies to enhance generalizability, and perform randomized controlled trials to test the efficacy of attitude‐modification interventions against traditional knowledge‐based approaches.

### 4.6 Policy and Practice Implications

These findings have immediate and actionable implications:

- **At the university level**: Health promotion must evolve beyond informational campaigns. Curricula, especially in health sciences, should integrate behavioral science to teach risk assessment, cognitive bias awareness, and stress management. The consistent international finding that knowledge acquisition correlates with increased self‐medication demonstrates a clear and urgent need for this pedagogical evolution [4, 13]. Peer‐led programs targeting social norms and the use of this study's predictive model to screen and support at‐risk first‐year students are recommended.
- **For public health agencies**: Campaigns targeting young adults should use narrative‐based communication and real‐world case studies to enhance risk perception and challenge favorable attitudes toward casual medication use.
- **At the regulatory level**: Policies that encourage pharmacist–patient interaction at the point of sale for OTC analgesics could serve as a crucial checkpoint, promoting safer use and encouraging professional consultation when necessary.

## 5 Conclusions

This investigation reveals an alarming prevalence of analgesic self‐medication among Ecuadorian university students, identifying it as a critical public health challenge. The study's central finding—that attitudes, not knowledge, are the primary driver of this behavior—necessitates a paradigm shift in health education and intervention. Substantiated by converging evidence from international research, the “knowledge–attitude paradox” among health sciences students underscores the insufficiency of traditional, information‐focused educational models and represents a systemic challenge for health education worldwide. Effective interventions must be grounded in behavioral science, aiming to reshape attitudes, enhance risk perception, and build decision‐making skills during the formative early years of university. The predictive model developed provides a valuable tool for identifying high‐risk individuals for targeted support. Ultimately, educational institutions and public health bodies share a responsibility to cultivate not just knowledgeable graduates, but also wise, self‐aware individuals who can serve as credible role models for responsible health behavior in their communities.

## Author Contributions

**Sonia Argote**: conceptualization, methodology, investigation, writing – original draft, project administration. **Doménica Bucheli**: investigation, data curation, writing – review and editing. **Ashel Páez**: investigation, formal analysis, visualization. **Camila Rovayo**: investigation, resources, data curation. **Daniel Freire**: investigation, validation, methodology. **Pablo Pila**: formal analysis, software, visualization. **José Daniel Sánchez**: conceptualization, methodology, writing – original draft, supervision, project administration.

## Funding

The authors have nothing to report.

## Ethics Statement

The study protocol was approved by the Institutional Review Board of Universidad Tecnologica Indoamerica (Protocol #UTI‐IRB‐2025‐001) and adhered to the Declaration of Helsinki. Anonymity and data confidentiality were maintained throughout the study.

## Consent

All participants provided written informed consent.

## Conflicts of Interest

The authors declare no conflicts of interest.

## Data Availability

The anonymized dataset supporting the findings of this study is available from the corresponding author upon reasonable request.
